# Between Depression and Alienation: Burnout as a Translator Category for Critical Theories

**DOI:** 10.1007/s11013-025-09929-0

**Published:** 2025-07-21

**Authors:** Domonkos Sik

**Affiliations:** Department of Social Theory, University of Eötvös Loránd, Pázmány Péter sétány 1/a, 1117 Budapest, Hungary

**Keywords:** Burnout, Critical theory, Emancipatory praxis, Depression, Alienation

## Abstract

The horizon of critical theories and their target audience (i.e., the subjects exposed to social suffering) have drifted apart. While the former relies on its own set of diagnostic concepts (e.g., alienation), the actors are socialized within the frames of biomedical discourses. This creates a rupture between theory and praxis: the concepts of social suffering fail to orient collective action. To overcome this challenge, a translator category is elaborated, which can link the distinct biomedical and critical discourses, while reconnecting theory and praxis. Burnout is chosen as a translator category because it is located at the border of the psychological discourses (on depression) and the critical sociological discourses (on alienation). First, the birth of the concept is reconstructed in a genealogical fashion. Second, the psychological measurement tools and explanations are overviewed with a special emphasis on the failed attempt of discursive medicalization. Third, the sociological explanations are analyzed from the perspective of their potential of breaking the biomedical and psychological discursive hegemony. In the last section, it is discussed how burnout can link the discourses of alienation (as a cause of burnout) and depression (as a consequence of burnout), while remaining accessible to the biomedically socialized subjects.

## Introduction

Critical theories have a hard time when it comes to translating their ‘diagnoses of the times’ (Hammershøj, 2014) into the everyday language of those actors who suffer from structural paradoxes (i.e., their target audience). While critical theories rely on their distinct set of concepts (such as alienation), the everyday actors are socialized within the frames of biomedical discursive power structures describing suffering with biomedical terms (such as depression—Sik, 2024a, 2024b). This creates a rupture in the anticipated trajectory of critical theories. They expect their diagnoses to orient emancipatory praxis: by reinterpreting individual experiences of suffering as consequences of structural paradoxes, the diagnoses are supposed to motivate actors to transform the distorting setting. However, the hegemony of biomedical discourse undermines this process: by being labeled as ‘ill’ or ‘mad’ (or clinically depressed), the sufferers are stuck in the realm of individualizing attributions, while also being deprived of agency. As illness and psychopathology become the predominant language of suffering, deservingness and solidarity also reconfigure: mainly the ill are entitled to social support (not the structurally deprived or the oppressed), while the main paradigm of support becomes health care intervention (not structural reform or social policy intervention). In this setting, critical theories expect in vain that the biomedically framed experiences of suffering will ground the motivational basis for transformative emancipatory praxis.

This deadlock is the starting point of my article. To overcome it, critical theory must turn its attention to concepts, which can connect the predominant biomedical discourses of suffering and the emancipatory discourses of critique. Because concepts such as depression and alienation are not evidently connectable, ‘translator categories’ are needed, which can bridge the distance between biomedical discourses (defining scientifically validated—i.e., objective thus deserving—suffering) and emancipatory discourses (describing the structural causes of suffering). Through such translator categories, the biomedical discourses (internalized by the lay actors) and the semantics of critique motivating emancipatory praxis can be synthesized. In other words, it can be ensured that the sociological diagnoses of times are perceived as accessible, relevant, and plausible because they refer to categories of illness without reducing actors to the objectifying role of the ill.^1^ My article explores such a translator category, the concept of burnout. Being equally linked to the discourses of depression and alienation, burnout promises to provide a phenomenologically accessible and critically reflective framework for interpreting social pathologies.

In the first section, the discursive ambivalences of burnout are explored in a genealogical fashion. Burnout was born in a special work environment, it was first observed among volunteers and human service workers (e.g., medical or nursing staff). Later, the general notion of ‘occupational burnout’ was elaborated (Heinemann & Heinemann, 2017). According to the current WHO definition, the symptoms of burnout are constituted of three interrelated dimensions: a sense of exhaustion; the distancing from one’s previously preferred job, negative or cynical work-attitudes; and reduced productivity and efficiency (WHO, 2019). Since empirical measuring tools have been developed, burnout was reluctantly embraced by the biomedical discourses. Although there are still strong critical voices arguing that burnout is merely a specific subtype of clinical depression (e.g., Bianchi et al., 2015), the category resists the full integration into biomedical discourse. The main cause of that resistance is the undeniable social component: burnout is inseparable from the world of labor, both on the level of symptoms and causes (Maslach et al., 2001). In this sense, burnout discourses remain ambivalent: they can neither define burnout exclusively as a psychopathology, nor interpret it as an organizational dysfunctionality. It can be used as an alternative of biomedical labels (e.g., depression) in those constellations, where psychopathologies are stigmatized. Or alternatively, it can be used as a proxy-medical category: if other diagnostic criteria are not met, burnout can be used as a justification for health care and social compensation (Schaufeli, 2017).

In the second and third sections, this under-defined notion is analyzed from the perspective of two rival discursive frameworks: the biomedical and the sociological. While the former approach elaborates diagnostic tools and tries to integrate the concept into the broader psy-discourses (often as a version of depression), these attempts do not completely succeed. Burnout contradicts several ontological and epistemological presumptions of the therapeutic discourse: it is partly caused by the workplace, so it cannot be explained or managed on a strictly individualistic basis. This ambiguity provides an opportunity for sociology to complement the biomedical burnout discourses. While there is little empirical research exploring structural factors, the mainstream sociological discourse on burnout operates on the level of ideology-critique and diagnosis of the times. According to these analyses, burnout can be primarily understood from the perspective of alienation. It becomes a general experience because the actors are constrained by the extending pressure of achievement (Ehrenberg, 1991; Petersen, 2024), acceleration (Rosa, 2019), and limited self-appropriation (Jaeggi, 2014).

Despite these efforts, critical sociology fails at challenging the biomedical hegemony of burnout discourses. So far it has missed the chance of transforming burnout into a translator category, which can facilitate emancipatory praxis. Based on this conclusion, in the last section, an attempt is made to reinterpret burnout: on the one hand, it is the consequence of alienating structural constraints; on the other hand, it has the potential of turning into depression (if the alienating constraints persist). By linking alienation and depression through burnout, the translation between biomedically socialized actors and the diagnosis of critical theory becomes possible, and emancipatory praxis is facilitated. 

## The Genealogy of Burnout

The discursive classification of people follows peculiar logic: although the expert categories seem to express ‘objective’ characteristics, as they are applied, they begin to interact with the classified actors, which changes the categories themselves (Hacking, 2006). The same dynamic can be observed in the case of burnout: first, the phenomenon is observed and initially described; second, measurement tools are developed to allow systematic analysis and intervention; third, the newly produced expert knowledge begins to influence public perception, so that lay actors interpret certain patterns of ‘unhappiness’ with the emerging diagnostic categories; fourth, such identification reconfigures the ‘objective’ features of burnout. This ‘looping effect’ explains why categories such as burnout must be explored in a genealogical manner: analyzing only their current form is misleading because it refers to an institutionalized, naturalized concept. With the help of genealogy, however, burnout can be understood as a continuously transforming category shaped by structural, discursive, and phenomenological factors (Foucault, 1977).

While the concept was born in the early 1970s, the historical antecedents of burnout can be traced back to pre-modern times, when similar experiences were described as *melancholia* or *acedia* (Schaffner, 2017). The next chapter in the history of exhaustion discourses was opened by the concept of *neurasthenia*, which has been described in the second half of the 19^th^ century as a disease originating from a modern lifestyle (Kury, 2017). Neurasthenia is a liminal category: it was born at a time when modernization was already having a noticeable impact on everyday life, while at the same time the psy-sciences had not yet become hegemonic. The key symptom of neurasthenia was diffuse physical and mental fatigue, which is inexplicable by organic causes (accordingly, one of the main therapies was resting). In the first half of the 20th century, neurasthenia as a diagnostic category was eclipsed: not because the phenomenon disappeared, but rather because of a change in psychological nosology. With the development and dissemination of diagnostic manuals, the diagnosis of other neurotic pathologies increased in proportion to the disappearance of neurasthenia (Taylor, 2001). In this sense, neurasthenia was gradually transformed into other, biomedically more elaborated categories.

The expansion and hegemonization of psychological and psychiatric discourses have been an ongoing process since the 20th century. This might make us wonder, how is it possible that after a long period of latency, in the 1970s, once again a similarly liminal, under-defined category emerges and gradually gains popularity in the public sphere? The circumstances of the ‘discovery’ of burnout are telling: Freudenberger coined the term based on his experiences in the ‘free clinic’ movement (1974). He observed that the behavior of many—initially very ambitious—volunteers followed a similar downward spiral. Firstly, the initial enthusiasm was replaced by an increasing sense of fatigue, which also manifested in various symptoms (e.g., headache, insomnia). In parallel with the somatic symptoms, the behavior also changed: a combination of impatience, overly suspicious (or even paranoid) thoughts, oscillation between remorse and a sense of omnipotence, risk-taking, stubbornness, inflexibility, and cynicism became a dominant pattern. The affected volunteers became increasingly isolated and were often considered depressed. Often, they forced themselves to improve (e.g., by volunteering more hours), yet they became less and less effective (Freudenberger, 1974).

According to this early analysis, burnout can be understood from the perspective of the dedication of the volunteers. They devote themselves to a good cause, which realistically cannot be resolved. However, they try harder and harder (both because of the internal moral obligation and the external recognition for the voluntary work), to the point of completely exhausting themselves. To reduce the harm caused by burnout, the volunteers were instructed to distance themselves from their activity. However, this process was made difficult by the initial devotion: because the volunteers’ identity and social network were organized around the good cause, distancing from the related activities was experienced as a loss. Accordingly, besides a sense of grief, also a sense of purposelessness and social disembedding was experienced, which would require special support (Freudenberger, 1974).

Although this early, first-hand description of burnout is rather intuitive and lacks systematic conceptual and methodological analysis, it still describes the main phenomenological components. The starting point of burnout is not simply a stressful or overburdening work environment: the internalized devotion for a certain value or goal also plays a central role. In those occupations, where one’s identity is at stake, failure is not an option. The subjects push themselves beyond their capacity, which manifests as internally motivated self-exploitation. Such processes are particularly difficult to reflect upon from the inside: while the exploited workers can clearly see that their exhaustion is the consequence of external constraints and coercion (i.e., the technologies of surveillance and discipline), the self-exploiting actors cannot see any obvious source of oppression. Because they internalize the drive to perform better, they have no one to blame for overburdening. This becomes a dangerous constellation, if the internalized devotion is paired with an infinite workload (e.g., in the context of voluntary work at a free clinic, this means the endless line of clients): the actors following their own devotion instead of external imperatives cannot set boundaries for themselves. While the original context of voluntary work might seem overly specific, reconstructing this genealogical background is indispensable for understanding how burnout became a more and more general experience.

Some might argue that the spread of burnout can be explained primarily as a discursive fashion: being an expressive metaphor, while also lacking clear scientific definition (there are currently more than a hundred definitions suggested by various experts), the concept is destined to spread in the public sphere (Hillert et al., 2020). However, I propose an alternative explanation, one that focuses on more fundamental, structural transformations. To understand the parallelism between the phenomenological horizon of volunteers and contemporary employees in general, the historical transformations leading from the ‘old’ to the ‘new spirit of capitalism’ needs to be evoked. According to Weber’s classic analysis, the pre-capitalist Protestant entrepreneur is primarily motivated by a religious work ethics: they do not want to accumulate wealth for the sake of consumption, influence or competitive advantage, but for the sake of handling their ‘salvation panic.’ In this context, working in a self-exploitative manner is an evident strategy: because worldly success indicates salvation, working toward wealth accumulation—even beyond one’s capacity–is an understandable behavior. In this context, any sort of occupation has a transcendental dimension in a sense that salvation depends on its success (Weber, 1992). From the perspective of burnout, the Protestant entrepreneur is just as much vulnerable as the contemporary volunteers: both of their activities are characterized by existential stakes, which has the realistic implication of self-exploitation.

With the expansion of capitalist markets, the world becomes ‘disenchanted,’ and the Protestant ethical component of entrepreneurship becomes marginal. Contemporary capitalism is a self-sustaining system, the ‘old spirit of capitalism’ loses its importance, while the structural constraints become decisive. Yet, at the end of the 20^th^ century, the spirit of capitalism becomes once again relevant: within the structural frames of the global, network capitalism, work ethics are reconfigured along the lines of the changing market constraints. It is in this context that the new spirit of capitalism is born: it provides the existential justification for the new *modus operandi* (Boltanski & Chiapello, 2005). The concept of the ‘market personality’—i.e., the predecessor of the subjectivity shaped by the new spirit of capitalism—was already described in the mid-20th century, as more and more actors identified themselves as commodities and measured their own worth by the possessions they owned (Fromm, 1955). As the new global markets demand extreme flexibility, real-time availability, and connectivity, this self-commodification has reached a new level. Gradually a new ideal of personality was born: the contemporary employee is supposed to become a hyper-flexible, multitasking, self-realizing entrepreneur, who runs parallel projects to create their own authentic life. According to this ideal, it is a life-long, individual quest to cope with the otherwise fragmented, deinstitutionalized life (Boltanski & Chiapello, 2005). Success has existential stakes as one’s worth depends on the ability to juggle with the overwhelming, unpredictable, and infinite flow of tasks. In this sense, contemporary subjects not only identify as commodities and owners but also as multitasking, enthusiastic self-entrepreneurs, who willingly exploit themselves.

However, such reconfiguration of the self is not without dangers: contemporary subjects are susceptible to the risk of ‘emptying.’ The new spirit of capitalism threatens to reproduce a fundamentally meaningless existence: the never-ending pursuit of the ideal of a non-stopping, multitasking agency ultimately undermines self-identity. An ‘empty self’ is born, which is characterized by a special need: to counter existential meaninglessness by seeking commodities in the market of experiences (Cushman, 1990). Advertising and psychotherapy exemplify the two ideal-typical counterstrategies provided by contemporary capitalism (Morris et al., 2020). Meaninglessness is either overcome through consumerism (including material goods and services grounding self-identities) or through therapeutic interventions (providing specialized services for those who fail to overcome meaninglessness by consuming—Sugarman, 2015). Such a project-based subjectivity—dependent on consumerism and therapy—is particularly susceptible to burnout.

As the management of the overwhelming parallel projects (within the realm of work and private life) becomes the basis of their—otherwise empty—self-identity, the contemporary actors become motivated to over-exploit themselves. Their existence is tied to the performance of work, but in a way that differs from the dedication of contemporary volunteers and early Protestant entrepreneurs. The actors following the ideals of project-capitalism have no choice but to take responsibility for themselves through succeeding in the realm of never-ending projects, while being consoled by consumption or therapy. Being constrained by existential structures based on unrestrained internal motivation, these project-based subjectivities are exposed to the risk of over-exhausting themselves (Petersen, 2024). Although they neither volunteer for a noble cause nor follow religious work ethics, the actors shaped by contemporary project-capitalism are in an existential trap. Their identity, social networks, and sense of worthiness are anchored in successfully dealing with an infinite flow of tasks. Because they are supposed to be exclusively responsible for their existence among the unpredictable, often precarious structural constraints, they have no choice but to do anything they can to prove their worth. However, while trying to do so, they exhaust themselves, and fail to find the hoped-for existential meaningfulness, which ultimately leads to burnout.

Despite its inherent relation to the constraints of contemporary capitalism, according to comparative analyses, the notion of burnout has divergent reception in various countries. While in the United States, it remained a term referring to a specific occupational disorder, in Europe (especially in Germany and the Scandinavian countries), it became a term expressing a pathological *Zeitgeist* (Heinemann & Heinemann, 2017). This difference has several implications, which are interrelated both with the cultural context and the moral economics of work. In the United States, project-capitalism is taken for granted as a natural structure of social existence. Accordingly, the burnout discourse is embedded in a more general discourse on self-responsibility and achievement. The neoliberal myth of the ‘self-made man’ naturalizes the devotion for one’s work (Boltanski & Chiapello, 2005). It is also taken for granted that such devotion might induce dynamics of over-exploiting the self, which eventually result in burnout. However, this framing also implies that burnout is not the end of the story, it is rather another challenge to be overcome. As much as burnout ‘proves’ that one has made every effort to achieve the best possible results, fighting burnout is the next logical step in the same trajectory. It ‘proves’ that one can defeat their own limitations: even the ones originating from over-exploiting themselves.

In contrast, the European burnout discourse is embedded in the context of a more critical relation to the overly accelerated and exploitative lifestyle originating from the new structures of capitalism. From this perspective, burnout is viewed as the ultimate consequence of the structural paradoxes, not as a necessary risk to be taken. It is not a natural hazard of the self-made man, but rather the negative consequence of forcing an overly exhaustive rhythm of work (Heinemann & Heinemann, 2017). Accordingly, it is not the individual who is responsible for overcoming burnout, but rather the social security system, and more specifically, the healthcare institutions. Because burnout is treated as a reason for sick leave, the need for its integration into the biomedical complex increases (Schaufeli, 2017).

In sum, burnout was born as a specific form of social suffering, which is not simply the consequence of an externally forced, overburdening job, but rather the consequence of being immersed in activities, which have existential stakes and uncertain success criteria at the same time. Among these activities, we can find the lifestyle of the religious-obsessive Protestant entrepreneur (driven by salvation panic), the contemporary volunteers and activists (driven by the identification with a good cause) and those subjects, who are entangled by project-capitalism. This last group constitutes the mass base for contemporary burnout discourses: because the ‘project-subjectivities’ believe that the cost of achieving autonomy and recognition is the adjustment to the requirements of network capitalism, they are immersed in multitasking. Since the demands of network capitalism are infinite, its structural constraints reproduce the susceptibility to burnout. While the emergence of project-capitalism explains the structural basis of burnout, it does not predetermine the related interpretations. To explore the rival discourses surrounding the under-defined phenomenon of burnout, both a psycho- and a social pathological parallel need to be compared. By clarifying the links between both burnout-depression and burnout-alienation, an attempt can be made to translate between these disciplines, which helps to broaden the horizon of potential countermeasures.

## Burnout vs. Depression: A Failed Discursive Takeover Attempt

Since the birth of the concept, burnout was viewed suspiciously by the biomedical sciences. It was mainly criticized for lacking clear and definitive diagnostic criteria, which prevents any further systematic analysis. This situation has only changed with the development of standardized measurement scales. The first and probably best known is the *Maslach Burnout Inventory*, which differentiates between the dimensions of emotional exhaustion, depersonalization, accomplishment, and optional personal involvement (Maslach & Jackson, 1981). The different variants of standardized burnout measurement tools combine these components with additional ones (e.g., Besèr et al., 2013; Kristensen et al., 2005). Although there are many debates about the internal and external validity of the scales, their creation opened the path for systematically analyzing the etiology and the medical consequences of burnout.

When it comes to the causes of burnout, the biomedical discourse is far from providing detailed models. Although biological research is relatively rare, according to the existing literature, the emergence of burnout is interrelated with the overstimulation of the nervous system, changes in certain hormonal levels (e.g., cortisol, that is the ‘stress hormone’), and decreased immune functions (Bayes et al., 2021). When it comes to the medical consequences and comorbidity of burnout, the literature is also cautious (due to the lack of specific biomarkers): memory problems, decreased brain activity (Grossi et al., 2015), immune-suppression, metabolic, and cardiovascular diseases, and ultimately premature death are among the most important consequences (Bayes et al., 2021; Kakiashvili et al., 2013). Although remaining inconclusive, this growing body of medical research reveals an important tendency: the attempt of fully integrating burnout into biomedical discourse. Despite the lack of definitive biomarkers, based on questionnaire-based diagnostic tools, wider and wider research is being conducted within the frames of medical sciences. Medical research is complemented by psychological analyses as well. On the level of personality traits, burnout is more frequent among people characterized by lower levels of hardiness (i.e., readiness to change), external attribution of control, passive and defensive coping strategies, lower self-esteem, competitiveness, neuroticism, emotional instability, and high job-related expectations. Besides these traits, the contextual impact is also central: it is not simply a specific personality type, which is susceptible to burnout, but rather the mismatch of certain work conditions and personality types (Maslach et al., 2001). According to positive psychology, the emergence of burnout can be understood from the perspective of its opposite, as the erosion of engagement (Maslach & Leiter, 1997). 

Medical and psychological research indicates the attempt of the biomedical discursive structures to integrate burnout into their framework. Perhaps the most radical of these attempts is to subordinate burnout to the already established conceptual framework of clinical depression. Since the early phases of the burnout discourse, depression was a conceptual rival. According to the most vehement critiques, burnout is either an overly vague, unscientific metaphor advocated by ‘pop-psychology’ or simply a subtype of depression. A growing body of research argues that not only do the symptoms of depression and burnout correlate strongly but also several background variables (such stressful life events, job adversity, and workplace support) overlap (Schonfeld & Bianchi, 2016). This means not only that the majority of actors diagnosed with burnout also meet the diagnostic criteria for depression but also that the characteristics of burnout can be explained as atypical depression. This approach is supported by the strong correlation between exhaustion (that is the most significant component of burnout) and symptoms of depression (Bianchi et al., 2014). From these observations comes the logical conclusion of getting rid of the biomedically inconsistent, scientifically unreliable category of burnout (Bianchi et al., 2015).

At this point, it is worthwhile to make a brief phenomenological comparison between depression and burnout. The lived experience of depression is constituted of several parallel dimensions: the inability to live through positive emotions; being stuck in an aching, powerless body; detachment from the mind, body, and the world; lost sense of purpose and hope; incompatibility of the past self and the depressed self; a sense of incarceration; lost control over thoughts and agency; feelings of numbness, emptiness, non-existence, and death (which is often paired with death wish); altered biorhythms; modified time consciousness (including the stagnation of the present, unending guilt related to the past, lacking horizon of the future); and disrupted intersubjectivity (communication difficulties, loneliness, stigmatization—Fusar-Poli et al., 2023). These dimensions constitute a coherently negative horizon: instead of a space of possibilities, the world becomes a horizon full of obstacles. Within a world characterized by impasses and unchangeability, agency is also gradually lost and gives its place to helplessness (Ratcliffe, 2015). The final component of a depressed lifeworld is narrowed interaffectivity: originally the other has the potential of dynamizing one’s affections; however, the depressed subjects cannot access these intersubjective impulses. They gradually become numb and ultimately isolated (Fuchs, 2013). Although most research focuses on internal bodily or mental causes, it must be emphasized that depression as a phenomenological pattern might also be internalized in a process of socialization. If the social environment is hindering, undermines the horizon of past and future, limits agency, and distorts intersubjectivity, depression might emerge as a form of social suffering (Sik, 2022, 2024a, 2024b). This social component—neglected by the mainstream biomedical depression discourses—helps to clarify the relationship between depression and burnout.

On a phenomenological level, burnout and depression are barely distinguishable: in both cases, the actors are detached from a world constituted of obstacles, while losing their agency and empathy. However, there is a difference in the attributed causes: most of the analyses that argue for a distinction from depression, despite the phenomenological similarities, refer mainly to the inherent social component of burnout. Even if on a phenomenological level, burnout is a universal potential, on a contextual level, it is inseparable from modernity and more specifically from the way work is organized in late capitalist societies (Schaufeli, 2017). Such focus on work has several practical consequences. On the one hand, for those who find the label of depression stigmatizing (because it implies an inherently ‘pathological’ body or mind), the label of burnout might seem to be acceptable substitute (as it refers to an external, overly exhaustive work environment). On the other hand, those who report burnout often do not see themselves as depressed, but rather as angry and frustrated. Their narrative is that they tried their best at a job that mattered for them, achieved certain goals, were exposed to overburdening pressure, collapsed mentally and somatically, and tried to get back on track (Engebretsen & Bjorbækmo, 2020). These actors do not interpret themselves as suffering from an inherent bodily or mental pathology but rather as failing to meet an overwhelming professional challenge.

In other words, the label of burnout enables different illness narratives than the label of depression: because the former is clearly related to a social context, it has the potential to shift the responsibility from the individual to the social. Of course, such narrative steps are not automatic: they require appropriate discursive frames, which can reorient the actors. These potential frames are explored in the next section, while synthesizing the sociological discourses on burnout and alienation.

## Burnout vs. Alienation: A Missed Opportunity for Critical Theories

The psychological discourse on burnout is marked by several ambivalences, which go back to the same ontological and epistemological ambiguity. First, burnout seems to fit in the psychological framework as a combination of affective (exhaustion, disinterest in work) and behavioral (cynical distancing, limited working capacity) components. However, from the moment of its birth, burnout resists the complete integration into the psychological discourse, because of the undeniable environmental component (stressful work conditions). Because burnout emerges in specific work settings, it is never only about the individual subject, but it is also about the workplace. On the one hand, this leads to an ontological question: should burnout be localized at the level of the individual (as a pathology of the subject) or the social (as a dysfunction of the institutional setting)? On the other hand, this implies an epistemological question: are the personality traits or the working conditions better indicators of burnout? Although these ontological and epistemological questions are explicitly not addressed by the mainstream burnout discourse, they are tacitly answered on a practical level, which is expressed by the chosen measurement tools. The most widely used burnout scales exclusively consist of questions about individual feelings of exhaustion, depersonalization or efficiency, while there are no questions about the features of the workplace. In other words, on an empirical level, the second dimension remains in the blindspot for most research, due to the one-sided measurement tools.

Although it is methodologically problematic to analyze a phenomenon that arises from both individual vulnerability and institutional dysfunction solely at the former level, this blindspot is seldom addressed, which expresses the hegemony of psychological discourse. The main terrain where the epistemological neglecting of the social dimension is contested is the practical level of intervention. Even if the predominant form of intervention remains counseling or psychotherapy, the reconfiguration of the working conditions is also included among the best practices since the birth of the concept^2^. According to the extended literature comparing the individual and the organization-based interventions, two consensual points seem to be crystallizing: the exclusively individualistic interventions can address only certain aspects of the burnout-syndrome, not its entirety (Ahola et al., 2017; Awa et al., 2010; Maricuţoiu et al., 2016); and the combination of the individualistic and organizational interventions is more effective than the ones operating either on this or that level (Aust et al., 2023; Maslach et al., 2001). In this sense, despite the unreflected ontological and epistemological ambivalence, the social component of burnout reappears at the pragmatic level. 

In order to explain why the social dimension is underrepresented despite its undisputable pragmatic relevance, the sociological discourses surrounding burnout need to be overviewed. Despite its increasing popularity in the public sphere, burnout did not get much attention from social sciences. According to the relatively little empirical research, the main social factors include younger age, unmarried family status, higher educational level (Maslach et al., 2001), extended work hours (Lim et al., 2010), role strain and role conflict related to bureaucratization (Varpio et al., 2018), job satisfaction (Tarcan et al., 2017), higher family status (Wu et al., 2022), irregular lifestyle, and limited social support (Huang et al., 2023). From these fragmented observations, the picture of an overly exhausting, unpredictable workplace and a precarious, yet educated structural position can be drawn as the main social risk factors. If burnout is generally understood as a mismatch of certain personality traits and social conditions, the latter component can be characterized more precisely with these features.

Besides these empirical results, there is a distinct, social theory-driven discourse of burnout. On the one hand, these analyses follow the logic of ‘ideology critique.’ According to this approach, burnout initially served a metaphorical function: it grasped a specific pattern of work-related suffering and enabled its public discussion. While gaining popularity, the metaphor was gradually inserted into the framework of expert discourses: it became an institutionalized entity, with its own measurement tools and institutional practices. Such institutionalization resulted in the reversal of the initial dynamics: instead of using burnout as a metaphor, lay actors are now shaped by the criteria elaborated in expert discourses. At this point, burnout serves ideological functions as the expert discourses differentiate between its relevant (i.e., the psychological vulnerability) and irrelevant dimensions (i.e., the institutional and structural constraints). By relying on expert discourses, lay actors also internalize individualistic attribution: they relate to their own experiences as psychological deficits or personal inadequacies, not as consequences of a dysfunctional work environment (Walker, 1986). The implication of such individualizing discourse is the depoliticization of burnout (Khan et al., 2023).

The other branch of social theoretical analysis of the ‘burnout society’ belongs to the tradition of diagnosis of the times (Han, 2015). Late modernity is characterized by a paradigm shift leading from ‘disciplinary society’ (based on the logic of surveillance and discursive governance—Foucault, 1995) to ‘achievement society’ (revolving around the constant pressure of performance—Ehrenberg, 1991). As the external forms of control are replaced by internalized mechanisms, the originally intersubjective dialectics of power become monological. Since it is the responsibility of the individual to control their own performance, the space for constant (self-)surveillance and (self-)constraining opens. While the Freudian subject was revolving around the confrontation with the external authority, the post-Freudian subject has no one to confront anymore (Han, 2015, p. 38). Because they are in constant competition with themselves and aim at triumphing over their own boundaries, they also become existentially isolated. As the originally intersubjective ‘struggle for recognition’ (Honneth, 1996) is transformed into a monodrama, the self becomes insecure and requires continuous reinforcement. This might either lead to narcissism, orpotentially depressive—self-loathing (if satisfaction cannot be reached), or the oscillation between these two phases. On a structural level, such existential vulnerability is ideal for contemporary capitalism, which exploits the constant desire for achievement, while providing painkillers for insecurity in the form of—addictive material (e.g., substances) and immaterial (e.g., information society)—consumption (Han, 2015, p. 51).

Although the critical theories working in the tradition of the Frankfurt School seldom address the question of burnout as a distinct phenomenon, they analyze a similar social pathology, namely alienation. The concept of alienation has developed in two parallel trajectories: the discourse started by Rousseau focuses on the tension between the individual and the social as such, while the Hegelian tradition reconstructs the conflict between the individual and certain alienating forms of the social (Jaeggi, 2014, p. 14). In the post-Hegelian era, Marx and Kierkegaard represented two alternative strategies for dealing with alienation: social criticism and revolutionary praxis aimed at overcoming alienation by social emancipation (Marx), while existentialism sought individual ways of escaping the alienating impact of social constraints (Kierkegaard). On a fundamental level, alienation is a ‘relation of relationlessness’ (Jaeggi, 2014, p. 25), a world-relation characterized by the impossibility of ‘resonance’ (Rosa, 2019). It does not simply refer to the limitation of one’s agency, or the deprivation of certain goods, but rather to a distorted modality of existing relationships. As alienation refers to a hiatus or an obstruction within the existing web of social ties, its overcoming can be understood as a process of reconfiguring one’s relation to the world.

The basic existential dynamic of inhabiting the world is appropriation: actors construct specific horizons of goals and become integrated into the world by achieving them. The success of appropriation is also the key to self-appropriation: the experience of autonomy and authenticity is inseparable from the ability to strive toward one’s own goals (i.e., the becoming of the subject through inhabiting the world). That is why the obstruction of appropriation is a decisive experience: it informs the subjects of their exposedness to external forces and ultimately their helplessness. Trying to achieve a certain goal in vain is the original experience of an alienated existence, controlled by external forces. It is also important that these external forces are often veiled (e.g., ‘false consciousness,’ ‘self-colonizing’ mindset); if they were not, that would enable the subject the possibility of circumventing them (Jaeggi, 2014, p. 201). This feature is responsible for the entangling aspect of alienation: the affected actors are not only obstructed in the process of (self-)appropriation but also they cannot access the sources of their hindrance, while being stuck in an existential impasse.

The links between alienation and burnout can be grasped at this point: the alienated actors are forced to exist in a structural constellation, which prevents them from self-appropriation, while also veiling the causes of the obstruction. An alienated existence is exhaustive: the continuous failed attempts of setting and realizing goals can be viewed as impossible, yet inevitable existential challenges. Alienating structures trap the actors: by hindering self-appropriation, they deprive them of the sense of fulfillment, autonomy and authenticity; by remaining veiled, they keep them in an endless loop of failed attempts at appropriation. The entrapped actors, seeing no better alternative, try harder: such futile efforts link the dynamics of burnout and alienation. Similarly to the Protestant entrepreneur and the contemporary volunteers and the employees shaped by project-capitalism, the alienated actors are also vulnerable to burnout, because they are destined to increase their performance without limitation, while being pressured by existential stakes. At this point, the theory of alienation appears as an important complement to the theory of burnout. It refers to a general vulnerability irreducible to the paradoxes of contemporary capitalism (Evans, 2022). The various causes of alienation, such as certain role systems (e.g., patriarchy), stigmas, and prejudices (e.g., ethnic stereotypes) can be as responsible for burnout as the existentially overburdening project-capitalism. 

Although critical theories elaborate their diagnoses of times at an abstract level, several empirical analyses reinforce their claims. If the boundaries of adequate performance become blurred, while the actors identify with the job, then a vicious spiral might emerge, which leads to the ‘collapse of the self-realization project’ (Thunman, 2012) or an ‘identity rupture’ (Korhonen et al., 2020). Numerous research seeks ways to implement sociological observations in emancipatory praxis: some argue that the increase of ‘civil, respectful social encounters’ (i.e., intersubjective encounters promising recognition) can decrease the probability of burnout (Maslach & Leiter, 2017); others advocate the importance of critical reflection in the workplace as a counter to blaming the employees for being over-burdened and exhausted (Mueller & Morley, 2020). According to similar research, criticizing and countering alienating structures has the potential to restore the collapsed project of self-appropriation, that is to create a less alienating—and a less burnout-prone— institutional environment.

Despite these theoretical and pragmatic conclusions, sociology somehow misses the chance of becoming a decisive voice in the expert and lay burnout discourses. The main reason for this is the mismatch between the ambition of sociology and the gaps in the discursive space. Sociology uses burnout mainly at the highly abstract level of ideology-critique or ‘diagnosis of the times,’ while it does not pay that much attention to an empirical analysis of the social causes and interventions. However, the main gap within the burnout discourses opens at the pragmatic level of organizational and institutional interventions. In this sense, what sociology provides is not the same as what the burnout discourses require. Consequently, the current theories of alienation seldom find their way to the expert and lay burnout discourses. These conclusions prepare the ground for answering our starting question: how can critical theories use the concept of burnout to facilitate emancipatory praxes?

## Burnout as a Translator Category: Enabling Emancipatory Praxis in the Biomedical Era

If critical theories hope to facilitate emancipatory praxis by revealing the structural paradoxes behind the individualized-medicalized horizon of suffering, they must adapt to the late modern discursive constraints. They need ‘translator categories,’ which can link the normative concepts implying social critique (such as alienation), with the hegemonic signifiers of suffering (such as depression)—burnout is an exemplary translator category in this sense^3^. Translation is fundamentally a hermeneutic process relying on the fusion of divergent horizons (Gadamer, 1989). Translator categories can be understood as facilitators of such a process: by providing mediating platforms that can be accessed from divergent perspectives, they contribute to the otherwise improbable mutual understanding. The category of burnout has such translational potential: it is open toward psychological and sociological directions, while resisting reduction to either of these discourses. Thus, it can help linking the psychologically attuned sufferers’ horizons with the critical theoretical diagnoses of the times. Because of its unique discursive position, burnout has the potential to expand the space of emancipatory praxis in the era of biomedical hegemony.

From a genealogical perspective, burnout expresses a contemporary version of existential impasse. It mainly affects those actors, whose identity revolves around a specific work-related activity: either because they identify with a good cause (e.g., volunteers), or because they internalized the ideals of project-capitalism (e.g., self-exploiting employees), or because they are trapped in an alienating constellation (e.g., victims of constraining latent structures), these actors push themselves beyond their capacity, while being driven by existential reasons (i.e., the desire for an autonomous, authentic or worthy life). Not only are they gradually consumed by their own efforts, but they are also prevented from perceiving the mechanisms and causes of their suffering. When exhaustion becomes unbearable, work becomes a distant and ultimately alien activity; by further forcing themselves, they risk bodily or mental breakdown. These transformative experiences require new interpretative schemas: the discourse on burnout answers this call by providing a medical, yet not necessarily objectifying framework.

Even within the biomedical discourse, the notion of burnout is located at the borders of diagnostic categories and metaphors of illness. Its ambiguous status provides an opportunity for experimenting within the given structural and discursive framework: depending on the context, if the illness label is the prerequisite of deservingness, burnout can be interpreted as a form of psychopathology; if the illness label is overly stigmatized (and the moral economy is permissive), burnout can be reinterpreted as a non-medical category. This ambivalence makes the category of burnout particularly adaptive: unlike the straightforward biomedical categories (such as depression), which deprive the subjects from their agency by reducing them to object-bodies and diagnostic labels, the partly scientific, partly metaphoric notion provides greater agency and autonomy. Furthermore, the failed medicalization of burnout is also explained by its inherent ontological ambiguities: not only does burnout lack specific biomarkers as diagnostic tools but it is also causally linked to structural constraints, which cannot be processed by the biomedical approach focusing strictly on individual (either bodily or mental) phenomena. Taken together, these factors explain why the relentless efforts to lump burnout into the broader category of depression continue to fail.

This discursive ambivalence provides a unique opportunity to use burnout for emancipatory purposes: because burnout cannot be fully integrated into biomedical discourse, there remains space for linking it to social critique. However, currently this opportunity is only partially exploited: the empirical sociological analyses of burnout are rare; the theoretical ones are either reduced to ideology-critique or elaborated as abstract diagnoses of the times. Although the mainstream concept of burnout partly serves an ideological function (by framing burnout as a mental burden, it obscures its institutional and structural causes), if reinterpreted in a critical manner, it can be used for revealing the alienating dynamics of contemporary capitalism. The newest theories of alienation open the path for such an interpretation: with their help it can be explained how burnout emerges in the futile yet inevitable struggle against the various structural constraints. If the appropriation of the world (and the parallel self-appropriation) is latently hindered by external forces, the subject is trapped in a perpetual, hopeless struggle, which might ultimately result in burnout.

All in all, burnout is in a unique discursive spot: being open to both medical categories (such as depression) and critical theoretical diagnoses (such as alienation), it provides an opportunity to link these discourses for lay actors. Instead of viewing depression as a discursive rival of alienation, the question should be raised: how can the concept of burnout establish a causal link between alienation and depression? The various forms of alienation (resulting from project-capitalism or alternative latent structures of subordination) pose an existential challenge: the actors are immersed in tasks that are existentially important but cannot be carried out in a reassuring way (e.g., the never-ending spiral of achievements or the never-ending struggle for self-appropriation). The longer one is exposed to such circumstances, the greater the likelihood of burnout: besides the raw exhaustion caused by the continuous pressure, the futility of the efforts leads to distancing from the immersing activities, which ultimately results in disillusionment and the collapse of (an impossible) self-identity. If one is forced to continue the alienating activities even after experiencing burnout, the likelihood of depression increases. The unchangeability of an unbearable existence reconfigures the horizon of expectations in several ways, which ultimately lead to depression: if self-fulfillment, resonance, and self-appropriation fail (i.e., alienating structures persist), the world transforms into a space of obstacles instead of opportunities; time loses its plasticity; agency is replaced by learned helplessness; and interaffectivity is replaced by isolation (i.e., a depressed phenomenological horizon emerges).

This distortive trajectory deserves the attention of critical theories: with its help, it could be explained how burnout arises from alienating structural constraints and turns into depression. According to the combined medical-sociological interpretation, burnout provides a rare example of an in-between phase of a complex process leading from a diffuse social suffering to a specific mental disorder (for further examples see Sik, 2022). By interpreting their condition through the metaphor of burnout, lay actors can recognize a need for intervention in an early phase, while avoiding medicalization. With the help of the combined medical and sociological discourses surrounding burnout, they might reflect on both its structural causes and mental health consequences. Furthermore, they can be motivated to prevent the emergence of depression by reconfiguring the distortive structures. Burnout is a unique translator category in this sense: it enables the biomedically socialized actors to see the medical consequences of a genuinely social suffering. In this sense, it has the potential of motivating emancipatory praxis, which directly counters the medically experienced (social) suffering, while indirectly reconfiguring the related paradox structures.

Although biomedical discourses aim at integrating burnout within their framework, for critical theories, it is particularly important to maintain its independent and ambivalent status. This ensures that those who are affected by an overly immersive activity (due to the fetishizing of achievements or the traps of alienation) could interpret their suffering in a non-medicalizing and non-objectifying fashion. Furthermore, this could ensure that the treatment of such existential impasses is not reduced to psychotherapeutic intervention but also includes the transformation of the social context. Exploring and expanding these practices is particularly important for critical theories, as these provide access points to the otherwise inaccessible horizon of those sufferers who are socialized within biomedical discursive frames. There is already a growing body of evidence about the importance of organization-based burnout interventions, which proves that there is an existing demand for sociological insight at this practical level. However, critical theories need to take a further step toward this direction: they must take burnout seriously as a translator category as well. By adjusting their conclusions to the specific requirements of those actors, who recognize their (social) suffering as burnout (potentially leading to depression), critical theories could actualize their currently untapped mobilization potential. By elaborating emancipatory interventions countering burnout, they could translate their diagnoses of times to the language of those sufferers, who could constitute the mass base for further structural reconfiguration. While the details of such a mobilization remain to be explored, the promise of overcoming the current impasse in critical theory and praxis makes it worth experimenting with.

## Footnotes

1 At his point, the similar ambitions of the ‘phenomenology of illness’ (Carel, 2016) or ‘mad studies’ (Russo and Sweeney, 2016) as counter-discourses should be mentioned: these approaches conceptualize illness and madness in a non-objectifying way, allowing affected subjects to retain their agency. My proposal advances these efforts by not only reframing an objectifying biomedical category but also linking it to critical theoretical discourses and emancipatory praxis.

2 Already in his programmatic article, Freudenberger suggests that modifying the time schedule should be used as prevention (1974).

3 Additional examples of such translator categories could be discovered through further analysis of popular psychology discourse (e.g., Lilienfeld et al., 2015).
